# Individual and context correlates of the oral pill and condom use among Brazilian female adolescents

**DOI:** 10.1186/s12905-021-01447-6

**Published:** 2021-08-19

**Authors:** Ana Luiza Vilela Borges, Luciane Simões Duarte, Alejandra Andrea Roman Lay, Elizabeth Fujimori

**Affiliations:** 1 Public Health Nursing Department, School of Nursing, University of São Paulo, São Paulo, Brazil; 2 School of Nursing, University of São Paulo, Avenida Doutor Enéas de Carvalho Aguiar, 419, São Paulo, SP 05403-000 Brazil; 3 Faculty of Health Sciences, University of Tarapacá, Avenida 18 de Septiembre, 2222, 1000000 Arica, Chile

**Keywords:** Female adolescents, Pill and condom use, Individual and contextual factors, Multilevel analysis, Brazil

## Abstract

**Background:**

Studies have examined the impact of contextual factors on the use of contraceptives among adolescents and found that many measures of income and social inequality are associated with contraceptive use. However, few have focused on maternal and primary health indicators and its influence on adolescent contraceptive use. This paper assesses whether maternal mortality rates, antenatal care visits, and primary healthcare coverage are associated with pill and condom use among female adolescents in Brazil.

**Methods:**

We used data from the Study of Cardiovascular Risks in Adolescents (ERICA), a national, school-based cross-sectional study conducted in Brazil. A subsample of all female adolescents who had ever had sexual intercourse and were living in one of the 26 State capitals and the Federal District was selected (n = 7415). Multilevel mixed effects logistic regression models were estimated to examine the effect of contextual variables on pill and condom use.

**Results:**

Sixty-five percent of female adolescents reported using pill while 21.9% reported using condom during the last sexual intercourse. Adolescents living in municipalities with low maternal mortality and high antenatal care coverage were significantly more likely to use pill during the last sexual intercourse compared to those from municipalities with high maternal mortality and low antenatal care coverage. Primary healthcare coverage (proportion of the population covered by primary healthcare teams) was not significantly associated with either condom or pill use during the last sexual intercourse.

**Conclusion:**

Our findings suggest that promoting the use of pill among female adolescents may require approaches to strengthen healthcare systems rather than those focused solely on individual attributes.

**Supplementary Information:**

The online version contains supplementary material available at 10.1186/s12905-021-01447-6.

## Background

Unintended pregnancies among adolescents remain a relevant public health challenge worldwide, especially in low and middle-income countries, where almost half of the pregnancies in individuals aged 15–19 years are estimated to be unintended [1]. In these countries, only one-third of sexually experienced females aged 15 to 19 use contraceptive methods [2].

There is substantial heterogeneity in adolescent contraceptive use across and within countries. However, adolescents mainly use short-acting methods, such as condoms and pills [1, 3–5]. Condoms are generally promoted among young people to prevent pregnancy and sexually transmitted infections (STIs), for example, HIV/AIDS. Condoms are widely available; providers often offer condoms instead of long-acting hormonal contraceptives (LARCs) because of a misconception that some LARCs are not appropriate for nulliparous women [6]. This situation may increase adolescents’ preference for condoms, at least in sporadic relationships, or at the beginning of new ones [7]. Several studies have demonstrated that the use of condoms during the last sexual intercourse differs among regions and countries and ranges from far less than 20% in many African and Latin-American countries [8, 9] to 74% in some European countries [10].

Worldwide, the prevalence of pill usage by adolescents is lower compared with condoms, ranging from 1 to 12% in Africa and South America, respectively [1], to 24% in Europe [10]. In general, female adolescents face many barriers to accessing oral contraceptives, because a common requirement to obtain them is a medical prescription, and specific policies and programs issued by governments limit girls’ access [6].

Cross-regional and -national differences in condom and pill use among adolescents have been explained by contextual factors, such as the sexual education programs at schools, availability of adolescent health services, and quality and access to family planning services and supplies [11]. Such contexts have often been ignored in investigations of adolescent contraceptive behavior, which have tended to focus on individual aspects, for example, age, socioeconomic status, behaviors such as alcohol use, and exposure to violence. Notably, the interventions that focus only on the individual level are likely to have a limited impact on contraceptive behavior [12]. Some studies that have examined the impact of contextual factors on the use of contraceptives have found that many measures of inequality, such as the Human Development Index and Gini coefficient, as well as poverty and investments in education, are associated with contraceptive use, especially condoms [13, 14].

Identifying contextual characteristics associated with individual practices could guide the implementation of effective family planning programs tailored to adolescent contraceptive needs [13]. Thus, our objective was to assess individual and contextual factors that influence the use of oral pill and condoms among Brazilian female adolescents. Our hypothesis is that female adolescents living in municipalities that present a better performance in some health indicators are more likely to use a condom or pill at the last sexual intercourse than their peers living in municipalities with inadequate performance on these indicators.

In the Brazilian context, 80% of adolescents report the use of contraceptive methods at the last sexual intercourse [15, 16]. While male condom was used by 69% of the adolescents, with no statistical difference by type of school (public/private) or region of residence, the use of pill was reported by only 13%, with significant differences between the regions [15]. Although male condoms are widely available in primary healthcare facilities, pills are available only with medical prescription in such contexts. The purchase of oral contraceptives without medical prescription is a common practice in the country, though not allowed. LARCs are rarely reported in Latin America and the Caribbean, including Brazil [17, 18].

## Method

### Study design

This study uses data from the Study of Cardiovascular Risks in Adolescents (ERICA). ERICA is a cross-sectional study carried out to assess the prevalence and covariates of cardiovascular risk factors in adolescents. It also assessed some other characteristics of adolescents’ lives, such as contraceptive practices.

### Sample and setting

The sample included male and female adolescents aged 12 to 17 years who attended public and private schools in 2014. The ERICA sampling design comprised all Brazil’s state capitals, the Federal District, and groups of other municipalities of more than 100,000 residents (large cities) from the macro-regions of the Country (North, Northeast, East-center, Southeast, and South). A sample of 1,251 schools was selected across all strata with probability proportional to size. In each school, three classes covering seventh to ninth grades in elementary school and from first through third grades in high school were randomly selected, and all adolescents from the selected classes were invited to take part in the research. Exclusion criteria and other details about the sample design are found elsewhere [19]. In total, 75,060 adolescents were included in the ERICA study.

The questionnaire for adolescents (available in English in Additional file 1) was self-administered using an electronic personal digital assistant [20].

We aimed to explore the individual and contextual influences related to the use of contraception at the last sexual intercourse. The focus was on female adolescents who had ever had sexual intercourse (n = 7415) living in one of the capitals and the Federal District.

### Procedures and variables

Multilevel logistic models with mixed effects were estimated to evaluate individual and contextual factors related to the use of the pill (n = 1629) or a condom (n = 4841) at the last sexual intercourse. Individual factors included age (12, 13, 14, 15, 16, and 17 years); skin color (white and non-white; non-white comprises respondents who self-reported as Black, mixed, Asian and Native Indian); family structure (living with parents, mother only, the father only, and none (i.e., can include other adult or a partner); mother`s education (low – from illiterate to less than 8 years of schooling; middle – from 8 to 11 years of schooling, high –12 or more years of schooling); type of school (public and private), paid jobs (no and yes), and age at sexual onset (until 14 years or 15 years and older).

Context variables were health indicators such as primary healthcare teams’ coverage, antenatal care coverage, and maternal mortality rate (MMR). Brazil is a country of continental dimensions with widespread regional and social inequalities. The country`s MMR has substantial regional disparities that reveal socioeconomic differences and inequalities in access to healthcare [21]. Regarding antenatal care coverage, there has been improvement in recent years, but there are still inequalities in access, with marked differences in the timing of the first visit and the number of visits across regions [22].

The variable primary healthcare coverage was measured by the percentage of the population covered by family health teams. A physician, a nurse, two nurse assistants, and four to six health community workers compose family health teams in Brazil, which is part of the Family Health Strategy. The Family Health Strategy was launched in 1994 to provide primary healthcare and improve health indicators in lower-income families. In 2014, the same year of our fieldwork, Brazil had approximately 39,000 family health teams: more than 265,000 community health workers serving 120 million people (62% of the population) [23]. Municipal-level analyses suggested that the strategy showed positive effects on health indicators, such as infant mortality, especially through the reduction of diarrhea- and pneumonia-related deaths [24]. Each health professional in the team has clear roles and tasks focused on responding to the most prevalent health problems and needs, including the provision of family planning services. The coverage of primary healthcare teams was calculated considering that each family health team is responsible for 3000 individuals residing in a geographical area close to a primary healthcare facility. The coverage of primary healthcare teams was analyzed in tertiles (1st tertile – municipalities whose coverage of primary healthcare teams was 27.4% to 50.7%; 2nd tertile – municipalities whose percentage was 52.3% to 64.0%; and 3rd tertile – municipalities whose percentage was 67.6% to 100.0%).

Within the range of reproductive health care, antenatal care ensures better maternal and infant outcomes [25]. Currently, high antenatal care coverage is captured through the number of visits [26]. The recommended antenatal care model of four visits was increased to eight visits for the entire period of pregnancy to guarantee increased opportunities to detect and manage potential problems [25]. In our study, high antenatal care coverage was measured by the percentage of newborns with more than seven antenatal care visits in 2013 – irrespective of whether the source of care was private or public services – because this is the minimum number of visits recommended by the Brazilian Ministry of Health [27] and this is how the information is available from the Information System on Live Births. We analyzed high antenatal care coverage in tertiles (1st tertile – municipalities whose percentage of newborns with more than seven antenatal care visits was 33.8% to 51.3%; 2nd tertile – municipalities whose percentage 64.6% to 70.2%; and 3rd tertile – municipalities whose percentage was 70.4% to 88.1%).

The monitoring of MMRs was relevant because MMR is an indicator of the performance of health services; thus, high rates are associated with poor quality of women`s health services, including family planning, antenatal, childbirth, and postpartum care [28]. The 2013 MMR of each capital was calculated from the Mortality Information System and the Information System on Live Births available at DATASUS (Information Technology Department of the Brazilian Public Healthcare System) (http://www2.datasus.gov.br/DATASUS/index.php?area=02). To calculate MMR, births and maternal deaths were adjusted using adjustment factors used by the Ministry of Health [29] to minimize the underreporting of maternal deaths. In Brazil, the MMR is high – approximately 60 maternal deaths per 100,000 births [29] – and unequally distributed among geographical macro-regions [30] due to inequalities in access to high-quality healthcare services, high rates of C-sections, and restrictive abortion laws. The MMR was analyzed in tertiles (1st tertile – municipalities whose MMR was 27.8 to 50.3 deaths per 100,00 births; 2nd tertile – municipalities whose MMR was 53.3 to 79.7; and 3rd tertile – municipalities whose MMR was 88.3 to 165.7).

Contextual factors were captured in 2013 and 2014, and pertain to the period before the interviews with students.

### Statistical analysis

All analyses were conducted using Stata^®^ software version 14.0 and took into account sample weights. We present the distribution of all female adolescents who had ever had sexual intercourse as well as those who used pill or condom during the last sexual intercourse by background characteristics.

We then estimated multilevel mixed-effects logistic regression models to examine the association between individual- and context-level factors and use of pill or condom during the last sexual intercourse, taking into account clustering of female adolescents at the municipality level.

We present (1) the empty model to obtain the variance attributed to the municipality, (2) the unadjusted models, (3) the association of all individual-level variables with each outcome (Model 1), and (4) the final model, that included both individual- and context-level factors. We present the crude and adjusted odds ratios with 95% confidence intervals (95%CI). Estimates with p-values less than 0.05 were considered statistically significant. We also computed the intraclass correlation coefficient (ICC), which provided the variation percentage of variation due to the contextual level, and proportional change in variance (PCV) of using the pill or a condom across the municipalities for Models 1 and 2. The PCV allowed us to assess the percentage change in the outcome variables attributed to the set of independent variables included in the models.

### Ethics

The Research Ethics Committees of each of the 26 states and the Federal District approved the study. Permission to conduct the study was obtained from all State and local Departments of Education and from all schools. All adolescents signed an informed assent form, and their parents signed a consent form.

## Results

### Characteristics of study participants

Adolescent females who participated in the study were mainly non-white, attending public schools, not working in paid jobs, and were living with their mother or both parents. Almost half had their first sexual intercourse at the age of 14 years or earlier. The majority reported using condoms (65.3%) at the last sexual intercourse, while only one out of five (29.1%) reported using pill (Table 1).

**Table 1:** Distribution of female adolescents who had ever had sexual intercourse, and used pill or condom at last sex by background characteristics

Variables	Ever had sexual intercourse (n = 7415)	Used pill at last sex (n = 1629)	Used condom at last sex (n = 4841)
	% (95% CI)	% (95% CI)	% (95% CI)
*Age*			
12	1.5 (1.2–2.0)	0.4 (0.1–1.1)	1.7 (1.2–2.3)
13	4.6 (4.0–5.4)	1.8 (1.1–2.8)	5.3 (4.4–6.3)
14	12.0 (10.8–13.2)	6.6 (4.9–8.7)	13.2 (11.7–14.7)
15	21.2 (19.8–22.7)	16.8 (13.9–19.8)	22.1 (20.1–24.1)
16	28.0 (26.4–29.5)	31.6 (27.6–36.0)	22.8 (26.2–29.4)
17	32.7 (31.0–34.4)	43.0 (37.3–48.9)	30.0 (28.4–31.9)
*Skin color*			
White	33.5 (31.4–35.6)	44.1 (39.0–49.4)	33.7 (31.5–35.9)
Non-white	66.5 (64.4–68.5)	55.9 (50.6–61.0)	66.3 (64.1–68.5)
*Type of school*			
Public	84.5 (78.8–88.8)	76.5 (63.3–86.0)	86.1 (82.5–89.0)
Private	15.5 (11.2–21.2)	23.5 (13.9–36.7)	13.9 (11.0–17.5)
*Worked in paid jobs*			
No	69.5 (67.7–71.2)	63.0 (58.6–67.2)	70.2 (68.0–72.2)
Yes	30.5 (28.8–32.3)	37.0 (32.7–41.4)	29.8 (27.7–32.0)
*Living arrangements with parents*			
Living with both	42.2 (40.5–43.7)	40.0 (36.5–43.6)	43.6 (41.8–45.5)
Living with mother	42.3 (40.7–43.9)	43.8 (39.4–48.2)	40.9 (38.8–43.0)
Living with father	4.6 (4.0–5.4)	4.9 (3.6–6.8)	5.0 (4.1–6.0)
Living with none	10.9 (9.8–12.2)	11.3 (8.5–14.8)	10.5 (5.2–11.9)
*Maternal educational level*			
Unknown	21.5 (19.2–24.1)	15.5 (11.3–21.0)	21.1 (19.1–23.1)
Low	21.1 (19.4–22.8)	18.8 (16.2–21.7)	22.0 (20.1–24.0)
Middle	39.1 (37.0–41.2)	42.4 (36.1–49.1)	38.1 (36.0–40.3)
High	18.3 (16.5–20.3)	23.2 (19.0–28.1)	18.8 (16.8–21.1)
*Age at first sex*			
Up to 14	49.3 (47.4–51.1)	44.3 (40.6–48.0)	47.6 (45.3–49.9)
15 or older	50.7 (48.9–52.6)	55.7 (52.0–59.4)	52.4 (50.1–54.7)

### Context-level characteristics

Brazilian State capitals are very diverse: population varied from 228,000 in Palmas, in the North, to more than 11 million in São Paulo, located in the southeast region. The coverage of primary healthcare teams varied from 27.4% in São Paulo to 100.0% in Vitória. Capitals with the highest proportion of antenatal care visits were Curitiba and Vitória (88.1% and 78.9%, respectively) while in Macapá and Porto Velho, only 33.8% and 34.6% of newborns had more than seven antenatal care visits. MMR varied from 27.81 deaths per 100,000 women in Palmas to 165.70 in Belém (Table 2).

**Table 2:** Characteristics of municipalities in Brazil, 2013–2014

Macro-regions	State capitals	Population 2010	Coverage of primary health care teams 2014 (%)	Newborns whose mothers made more than 7 antenatal care visits 2013 (%)	Maternal mortality rate (adjusted) 2013
North	Belém	1,393,399	45.5	62.5	165.70
	Boa Vista	284,313	67.6	50.7	42.30
	Macapá	398,204	83.9	33.8	112.62
	Manaus	1,802,014	52.3	41.6	90.30
	Palmas	228,332	82.2	63.0	27.81
	Porto Velho	428,527	61.9	34.6	105.30
	Rio Branco	336,038	91.7	48.2	56.20
Northeast	Aracaju	571,149	82.6	54.6	53.34
	Fortaleza	2,452,185	44.4	51.1	65.18
	João Pessoa	723,515	88.0	62.7	127.92
	Maceió	932,748	49.7	50.7	79.67
	Natal	803,739	64.1	57.1	56.70
	Recife	1,537,704	58.4	56.7	60.41
	Salvador	2,675,656	34.8	51.3	88.41
	São Luís	1,014,837	40.8	43.7	93.67
	Teresina	814,230	84.8	56.4	50.28
Southeast	Belo Horizonte	2,375,171	94.2	76.8	38.24
	Rio de Janeiro	6,320,446	50.7	71.7	76.43
	São Paulo	11,253,503	27.4	75.1	45.67
	Vitória	327,801	100.0	78.9	28.83
South	Curitiba	1.751.907	60.6	88.1	32.09
	Florianópolis	421,240	96.5	70.9	47.70
	Porto Alegre	1,409,351	69.1	74.1	40.83
Mid-West	Brasília^a^	2,570,160	55.4	69.5	72.39
	Campo Grande	786,797	61.4	69.1	88.27
	Cuiabá	551,098	52.4	70.3	29.63
	Goiânia	1,302,001	57.5	70.2	42.68

^a^Federal District

### Individual- and context-level factors associated with pill or condom use

There was a wide variation in the use of pill among female adolescents across capitals, ranging from 4.8% in Macapá to 47.9% in Porto Alegre (data not shown in table). Results from the fully adjusted model showed that MMR and antenatal care coverage were significantly associated with use of pill during the last sexual intercourse among female adolescents. At the municipality level, higher MMR was inversely associated with using pills while higher antenatal coverage was positively associated with use of the method during the last sexual intercourse. The coverage of primary healthcare teams was not significantly associated with using pills. At the individual level, older adolescents, non-whites, those working in paid jobs, those living with no parents, and those whose mothers had medium or high educational level were significantly more likely to use pill compared with the respective sub-groups of adolescents.

In the null model, the ICC showed that approximately 9.7% of the total variance on the use of pill was attributed to municipality level and 90.3% to individual level factors. The inclusion of individual-level variables explained 10.2% of the context-level variance (Model 1). In Model 2, the PCV showed that 64.9% of the variance at the municipality level were credited to the individual and contextual variables related to pill use (Table 3).

**Table 3:** Odds ratios from multilevel logistic regression analysis examining the association between individual- and context-level factors and use of pill at last sexual intercourse among female adolescents in Brazil, 2014

Variables	Null model	Unadjusted model OR (95% CI)	Model 1 OR (95% CI)	Model 2 OR (95% CI)
*Individual level*				
Age		1.33 (1.24–1.42)	1.33 (1.24–1.44)	1.34 (1.24–1.45)
*Skin color*				
White		1	1	1
Non-white		0.71 (0.61–0.84)	0.77 (0.65–0.90)	0.77 (0.65–0.90)
*Type of school*				
Public		1	1	1
Private		1.55 (1.18–2.02)	1.26 (0.96–1.66)	1.26 (0.96–1.66)
*Worked in paid jobs*				
No		1	1	1
Yes		1.26 (1.12–1.42)	1.21 (1.07–1.38)	1.21 (1.07–1.38)
*Age at first sex*				
Up to 14		1	1	1
15 or older		1.19 (0.99–1.43)	0.86 (0.68–1.07)	0.86 (0.68–1.07)
*Living arrangements with parents*				
Living with both		1	1	1
Living with mother		1.10 (0.78–1.56)	1.25 (0.87–1.80)	1.25 (0.87–1.80)
Living with father		1.06 (0.87–1.29)	1.09 (0.89–1.34)	1.09 (0.89–1.34)
Living with none		1.40 (1.04–1.87)	1.47 (1.10–1.96)	1.47 (1.10–1.96)
*Maternal educational level*				
Low		1	1	1
Medium		1.49 (1.17–1.91)	1.39 (1.07–1.81)	1.39 (1.07–1.81)
High		2.10 (1.53–2.87)	1.85 (1.32–2.57)	1.85 (1.32–2.57)
*Municipality level*				
*Primary health care teams coverage*				
1st tertile		1		1
2nd tertile		1.12 (0.69–1.81)		0.85 (0.57–1.27)
3rd tertile		1.31 (0.74–2.32)		0.85 (0.52–1.40)
*Antenatal care visits*				
1st tertile		1		1
2nd tertile		1.81 (1.19–2.76)		1.64 (1.20–2.21)
3rd tertile		2.97 (1.84–4.78)		1.89 (1.32–2.70)
*maternal mortality rate*				
1st tertile		1		1
2nd tertile		0.58 (0.42–0.79)		0.71 (0.52–0.98)
3rd tertile		0.38 (0.22–0.64)		0.55 (0.34–0.87)
Total variance (SE)	0.354 (0.118)		0.318 (0.104)	0.124 (0.037)
ICC	0.097			
Proportional change in variance (PCV)		10.2%	64.9%	

Model 1: adjusted for all individual-level variables. Model 2: adjusted for individual-level and municipality-level variables

The use of a condom ranged from 50.1% in João Pessoa to 77.5% in Macapá (data not shown in table). No contextual variable was significantly associated with use of condom in the unadjusted model. In the null model, the ICC showed that approximately 2.7% of the total variance in condom use was attributed to municipality level and 97.3% to individual level factors. Given such a low ICC, the full-adjusted model with the context-level variable was not estimated. Results from the model adjusted for individual-level factors showed that older adolescents, non-white, and those living with no parents were significantly less likely to use a condom, while those who had first sexual intercourse at age 15 or older were significantly more likely to use a condom compared with the respective comparison sub-groups (Table 4).

**Table 4:** Odds ratios from multilevel logistic regression analysis examining the association between individual- and context-level factors and condom use at last sexual intercourse among female adolescents in Brazil, 2014

Variables	Null model	Unadjusted model OR (95% CI)	Model 1 OR (95% CI)
*Individual level*			
Age		0.91 (0.86–0.96)	0.83 (0.77–0.89)
*Skin color*			
White		1	1
Non-white		0.80 (0.69–0.93)	0.83 (0.72–0.96)
*Type of school*			
Public		1	1
Private		1.32 (1.07–1.63)	1.11 (0.89–1.39)
*Worked in paid jobs*			
No		1	1
Yes		0.89 (0.78–1.00)	0.94 (0.82–1.07)
*Living arrangements with parents*			
Living with both		1	1
Living with mother		0.89 (0.69–1.14)	0.88 (0.68–1.13)
Living with father		0.87 (0.74–1.01)	0.87 (0.75–1.01)
Living with none		0.75 (0.61–0.92)	0.79 (0.64–0.97)
*Maternal educational level*			
Low		1	1
Medium		0.98 (0.72–1.33)	0.96 (0.70–1.32)
High		1.25 (0.97–1.62)	1.15 (0.85–1.56)
*Age at first sex*			
Up to 14		1	1
15 or older		1.35 (1.16–1.56)	1.61 (1.36–1.91)
*Municipality level*			
*Primary health care teams coverage*			
1st tertile		1	
2nd tertile		1.17 (0.91–1.51)	
3rd tertile		1.19 (0.90–1.57)	
*Antenatal care visits*			
1st tertile		1	
2nd tertile		0.80 (0.59–1.09)	
3rd tertile		0.84 (0.63–1.13)	
*Maternal mortality rate*			
1st tertile		1	
2nd tertile		1.00 (0.77–1.30)	
3rd tertile		1.10 (0.80–1.52)	
Total variance (SE)	0.091 (0.022)		0.100 (0.256)
ICC	0.027		

Model 1: adjusted for all individual-level variables

## Discussion

This study presents new insights regarding the association between health indicators and use of contraceptives among female adolescents from cities of a middle-income country with advanced primary health care services but high levels of social inequality [23]. Specific interventions to promote the use of contraceptives that have proven effective include training providers in adolescent contraceptive counseling, providing free or over-the-counter contraceptives for adolescents, and making health services adolescent or youth-friendly [31–33]. Our findings suggest that the context in which adolescents live also matter for use of certain contraceptive methods. The findings, for instance, show that living in a municipality with low MMR or a high number of antenatal visits is positively associated with use of pills among female adolescents.

Our findings are consistent with those of a European study which found that geographical differences in adolescent pill and condom use could be explained by diverse access to reproductive health services and contraceptive supplies [10]. Certainly, in countries where there is high MMR, there is low prenatal care coverage and limited access to contraceptives [34]. Consequently, general investments in maternal health may result in improvements in the uptake of some family planning methods among adolescents as initiatives dedicated to women in general, not adolescent-specific, seem to influence contraceptive usage.

Adolescents could therefore benefit from improvements in reproductive health services that expand availability and access to contraceptives in primary healthcare facilities. Access to pills, especially where there are restrictions on contraceptive methods available to adolescents, is likely to contribute to prevention of unintended pregnancies and associated adverse health and socio-economic outcomes among this subset of the population. This is especially relevant because worldwide trends show that adolescent fertility rates in Latin America are the second highest in the world [35] and that Latin America is the only region where unintended pregnancies resulting in birth, as a proportion of all births, have increased [36], with limited progress in reducing adolescent pregnancy in the region.

The consequences of unintended pregnancies among adolescents are even worse compared with adult women because the adverse social and economic consequences may overlap with the adverse health outcomes [37]. Although the pill is a short-acting method whose effectiveness is highly dependent on user self-discipline, which can be problematic to adolescents, it provides a safe and effective way of preventing unintended pregnancies when taken correctly [38, 39]. Given that oral contraceptives are mainly obtained at commercial drugstores in Brazil, it could be that investments in reproductive health services would not make a difference for adolescents’ access to the method. However, the purchase of oral contraceptives is more frequent among high- than low-income women [40], which means that most vulnerable adolescents still rely on primary healthcare services to obtain contraceptives, and are likely to take advantage of any enhancement on the quality of reproductive health services.

Although our findings show individual-level variations in condom use, much of the variation in use among the municipalities remains unexplained, which is inconsistent with findings from other studies which showed that contextual factors, such as gross domestic product, population growth and Human Development Index are correlated with condom use [13, 14]. This could reflect the fact that condoms are widely available in primary healthcare facilities in Brazil, along with other interventions concerning HIV/AIDS prevention, such as rapid testing and community-based health education programs. Besides being offered in primary healthcare facilities, pharmacies and drug stores, condoms are also available nationwide at low cost or even for free in a range of non-traditional outlets such as metro stations, grocery stores, supermarkets, convenience stores, nightclubs and during popular festivals, like carnivals. Context-level influence on adolescent condom use remains a gap to be addressed in future studies.

The male condom is effective against HIV and cost-effective for HIV control and prevention, but its consistent use for prolonged periods remains low, especially because of low-risk perception, non-intention of use, the need to negotiate its use with every partner, the belief that condoms reduce sexual pleasure, and use of alcohol and drugs [4, 7]. Furthermore, although our findings show that condom is used more frequently than pill, such use may be temporary and inconsistent. Literature shows that adolescents switch from condoms to pills or other contraceptive as they age and change relationships from casual to more steady and regular partners [41, 42]. The frequency of contraceptive transitions among adolescents suggest a need for health professionals to actively support the consistent use of condoms by adolescents whether during medical consultations or at schools, including dual contraceptive education, as condoms remain the best form of dual protection against STIs and unintended pregnancy.

Regarding the individual-level factors associated with contraceptive use, our results showed that older adolescents were more likely to use pill and they were less likely to use a condom than their younger counterparts. Literature shows that as adolescents age, they increase the use of effective contraceptives [43], switching from condoms to pill or other highly effective methods. These patterns suggest that efforts to provide sexual education to adolescents, including information on contraception, should reach them in their early adolescence to enable them make healthy and conscious decisions about their own sexuality.

Our findings show that non-white adolescents had lower probabilities of using contraception compared to white girls. The findings are similar to those of a study in the US which found low contraceptive use among Black and Hispanic adolescents [44]. Racial disparity in contraceptive use in Brazil reflects the vulnerability of black women to poor reproductive health outcomes, including obstetric complications and poor access to prenatal care [45, 46]. Tailored interventions to account for such disparities are a public health priority in the country.

Our findings further show that adolescents with highly educated mothers were more likely to use pill, compared to those whose mothers had low levels of education, while maternal education had no significant association with condom use. Highly educated mothers may be more aware of highly effective contraception and may have a better approach to communicating such issues with their daughters compared to those with no or low levels of education [47]. Great progress has been made in expanding access to basic education in Brazil, but not every woman has benefited from these advances. Socioeconomic status and education levels continue to play an important role in the access to sexual and reproductive health services, including contraception and abortion [48], and this suggests a need for policies and programs to address such inequalities in the country.

The findings of this study further show that adolescents who were involved in paid work, and those who reported living with no parents were more likely to use pill compared to those who were not involved in paid work or who lived with their parents. Adolescents involved in paid work and those living with no parents may comprise girls who have some autonomy in social life, which provides freedom in sexual and contraceptive choices, especially considering that pill is mainly accessed in retail drugstores. Given that adolescents face many barriers such as lack of information and prejudice at unfriendly health services [6], many of them who work and have their own money prefer to buy their contraceptives themselves [49]. Similarly, adolescents who do not live with parents may be married, i.e., in stable relationships, and with some economic self-independence, which may enable them access pills.

The availability of condoms is essential, particularly in HIV-endemic areas or regions where incidence is high among young people, and oral contraceptives remain an effective form of contraception even for adolescents. However, girls should still be given an opportunity to choose a method among a wide variety of contraceptives, including LARCs; thus, the method mix in Brazil should be expanded for adolescents [31] as it is limited to short acting methods [18].

## Limitations

Although a robust sample of nationally represented Brazilian adolescent females aged 12 to 17 years was used, the cross-sectional nature of the study does not allow making causal inferences regarding the relationship between the factors considered and pill use. Information concerning the partner, or their affective-love relationship, was not considered, but steady couples who use the pill might feel that condom use is unnecessary. Our main outcomes, pill or condom use, denote a fixed behavior reported from the last sexual intercourse, limiting the understanding of any trend of continuation or discontinuation of the method used. Additionally, our data was collected in 2014, but recent studies have shown that no significant changes in adolescent contraceptive use have occurred in Brazil [18]. However, contextual factors may have worsened due to the onset of the COVID-19 pandemic, with many disruptions to reproductive health services worldwide [50].

## Conclusion

Our findings show that sexually experienced female adolescents from municipalities with low maternal mortality and high antenatal care coverage were significantly more likely to use pill during last sexual intercourse compared to those from municipalities with high maternal mortality and low antenatal care coverage. However, variations in condom use were only evident at the individual but not contextual level. The findings suggest that promoting the use of pill among female adolescents may require approaches for health system strengthening rather than those solely focused on individual attributes. We posit that adolescents residing in municipalities with effective maternal healthcare systems are more likely to access family planning counseling and contraceptive supplies, especially the pill, compared to those from municipalities with ineffective systems.

## Supplementary Information

**Additional file 1.** Adolescents’ questionnaire.

## Data Availability

The datasets generated and/or analyzed during the current study are not publicly available due to regulations of the ERICA project. The data are available on request, which may be sent to alvilela@usp.br.

## Abbreviations

DATASUS: Information Technology Department of the Brazilian Public Healthcare System
ERICA: Study of Cardiovascular Risks in Adolescents
ICC: Intraclass correlation coefficient
LARC: Long-acting reversible contraceptives
MMR: Maternal mortality rate
PCV: Proportional change in variance
STI: Sexually transmitted infection
