# Supplementary material for: Individual and context correlates of the oral pill and condom use among Brazilian female adolescents
Source: BMC Womens Health. 2021 Aug 19;21:307. doi: 10.1186/s12905-021-01447-6 (PMC8374415; doi:10.1186/s12905-021-01447-6)
Supplement: Supplementary file 1 — Additional file 1. Adolescents’ questionnaire. [file 12905_2021_1447_MOESM1_ESM.pdf]

ADOLESCENTS' QUESTIONNAIRE

ERICA
Estudo de Riscos Cardiovasculares em Adolescentes

ERICA
ESTUDO DE RISCOS CARDIOVASCULARES EM ADOLESCENTES

Block 1: Socio-demographic factors

QUESTION	OPTIONS
What is your sex?	1. Female<br>2. Male
What is your skin color or ethnicity?	1. White<br>2. Black<br>3. Mixed races<br>4. Asian (yellow)<br>5. Indigenous<br>77. I don't know / I prefer not to answer
How old are you?	|__|__| years old
Do you live with your mother?	1. Yes<br>0. No
Do you live with your father?	1. Yes<br>0. No

What is your mother's schooling?	0. Illiterate/< 1 year<br>1. 1 to 3 years of study<br>2. 4 to 7 years of study<br>3. Completed elementary school<br>4. Incomplete high school<br>5. Completed high school<br>6. Incomplete college<br>7. Completed college<br>77. I don't know/ don't remember
Counting with you, how many people live in your residence?	|__|__| persons
How many rooms have in your residence?	|__|__| rooms
Counting with you, how many people sleep in the same bedroom or room as you?	|__|__| persons
How many televisions are there in your residence?	|__| zero |__| one |__| two |__| three |__| four or more |__| I don't know/ don't remember
How many radios are there in your residence (including integrated in another device)?	|__| zero |__| one |__| two |__| three |__| four or more |__| I don't know/ don't remember
How many bathrooms are there in your residence?	|__| zero |__| one |__| two |__| three |__| four or more |__| I don't know/ don't remember
How many cars are there in your residence for personal or family use (do not consider cars used for professional activities)?	|__| zero |__| one |__| two |__| three |__| four or more |__| I don't know/ don't remember
How many salaried domestic	|__| zero |__| one |__| two |__| three |__|

ERICA
ESTUDO DE RISCOS CARDIOVASCULARES EM ADOLESCENTES

workers work at your home permanently for five days or more per week, including nannies, drivers, etc?	four or more |__| I don't know/ don't remember
How many washing machines are there in your residence?	|__| zero |__| one |__| two |__| three |__| four or more |__| I don't know/ don't remember
How many videocassettes or DVD players are there in your residence?	|__| zero |__| one |__| two |__| three |__| four or more |__| I don't know/ don't remember
How many refrigerators are there in your residence?	|__| zero |__| one |__| two |__| three |__| four or more |__| I don't know/ don't remember
How many freezers are there in your residence? (do consider freezer or 2nd door in duplex-refrigerator)	|__| zero |__| one |__| two |__| three |__| four or more |__| I don't know/ don't remember
How many motorcycles are there in your residence (for personal or family use)?	|__| zero |__| one |__| two |__| three |__| four or more |__| I don't know/ don't remember
Is there any computer in your residence?	0. No<br>1. Yes, with internet access<br>2. Yes, without internet access
Who do you consider the head of your family?	1. My father<br>2. My mother<br>3. Other person<br>77. I don't know
Which is the schooling of your father?	0. Illiterate/< 1 year<br>1. 1 to 3 years of study<br>2. 4 to 7 years of study<br>3. Completed elementary school<br>4. Incomplete high school<br>5. Completed high school<br>6. Incomplete college<br>7. Completed college<br>77. I don't know/ don't remember
Which is the schooling of the head of your family?	0. Illiterate/< 1 year<br>1. 1 to 3 years of study<br>2. 4 to 7 years of study<br>3. Completed elementary school<br>4. Incomplete high school<br>5. Completed high school<br>6. Incomplete college<br>7. Completed college<br>77. I don't know/ don't remember

Block 2: Work

22. DURING THE LAST YEAR, did you work receiving payment in cash or some benefits? You can select more than one option.

1. I did not work.
2. As an employee (in shop/market, agriculture, raising animals, office, company…)?
3. As a trainee (in shop/market, agriculture, raising animals, office, company…)?
4. Self-employed/Odd Jobs (in shop/market, agriculture, raising animals, office, company…)?
5. In family home, doing housework (cleaning, cooking or organizing) or as a nanny?

22. DURING THE LAST YEAR, did you work without payment in cash or some benefits? You can select more than one option.

1. I do not work.
2. As an employee (in shop/market, agriculture, raising animals, office, company…)?
3. As a trainee (in shop/market, agriculture, raising animals, office, company…)?
4. Self-employed/Odd Jobs (in shop/market, agriculture, raising animals, office, company…)?
5. In family home, doing housework (cleaning, cooking or organizing) or as a nanny?

ERICA
ESTUDO DE RISCOS CARDIOVASCULARES EM ADOLESCENTES

23. In the last week you practiced:

Activity:	1. Yes<br>0. No	1. School<br>2. Outside of school<br>3. Inside and outside of school
a. Soccer (field, street, club)		
b. Indoor soccer futsal		
c. Handball		
d. Basketball		
e. Roller skate or skateboard		
f. Athletics		
g. Swimming		
h. Artistic or rhythmic gymnastics		
i. Judo, karate, capoeira, other fights lutas		
j.*Jazz*, *ballet*, modern dance, other types of dance		
l. Running, *jogging*		
m. Ride a bike		
n. Walking as a form of exercise		
o. Walk as a form of commuting to school, work or a house of a friend		
p. Volleyball		
q. Beach Volleyball		
r. Play catch, hide-and-seek, skipping hope		
s. Surf, *bodyboard*		
t. Bodybuilder in gym		
u. Abdominal exercises, push-ups or legs s		
v. Tennis		
x. Dog-walking		
y. Fitness gym (aerobic, localized)		
w. Beach soccer		
z. Taking care of children under 5 years old		

ERICA
ESTUDO DE RISCOS CARDIOVASCULARES EM ADOLESCENTES

Block 4: Diet

**24.** Do you eat the meal offered by the school?	|__| My school does not offers meal| __| I do not eat the meal offered by the school.<br>|__| I do eat the meal offered by school sometimes.<br>|__| I do eat the meal offered by school almost every day.<br>|__| I do eat the meal offered by school every day.
**25.** Do you buy snacks at the school's snack bar?	|__| I do not buy snacks at the school's snack bar.<br>|__| I do buy snacks at the school's snack bar sometimes<br>|__| I do buy snacks at the school's snack bar almost every day.<br>|__| I do buy snacks at the school's snack bar every day.<br>|__| My school does not have a snack bar.
**26.** Do you eat breakfast?	|__| I do not eat breakfast.<br>|__| I do eat breakfast sometimes.<br>|__| I do eat breakfast almost every day.<br>|__| I do eat breakfast every day.
**27.** Do you eat lunch watching TV?	|__| I do not eat lunch watching TV.<br>|__| I do eat lunch watching TV sometimes.<br>|__| I do eat lunch watching TV almost every day.<br>|__| I do eat lunch watching TV every day.
**28.** Do you eat dinner watching TV?	|__| I do not eat dinner watching TV<br>|__| I do eat dinner watching TV sometimes<br>|__| I do eat dinner watching TV almost every day.<br>|__| I do eat dinner watching TV every day.
**29.** Do your father (or stepfather) or mother (or stepmother) or guardian have lunch with you?	|__| My parents or guardians never or almost never eat lunch with me.<br>|__| My parents or guardians eat lunch with me sometimes.<br>|__| My parents or guardians

	eat lunch with me almost every day.<br>|__| My parents or guardians eat lunch with me every day.
**30.** Do your father (or stepfather) or mother (or stepmother) or guardian have dinner with you?	|__| My parents or guardians never or almost never eat dinner with me.<br>|__| My parents or guardians eat dinner with me sometimes.<br>|__| My parents or guardians eat dinner with me almost every day.<br>|__| My parents or guardians eat dinner with me every day.
**31.** Do you watch TV eating snacks like popcorn, cookies, sandwiches, chocolates or candies?	|__| I do not watch TV eating snacks.<br>|__| I do watch TV eating snacks sometimes.<br>|__| I do watch TV eating snacks almost every day.<br>|__| I do watch TV eating snacks every day.
**32.** Do you eat snacks like popcorn, cookies, snacks, sandwiches, chocolates or candies using the computer or playing video games?	|__| I do not eat snacks using the compute or playing video games.<br>|__| I do not eat snacks using the compute or playing video games sometimes<br>|__| I do not eat snacks using the compute or playing video games almost every day.<br>|__| I do not eat snacks using the compute or playing video games every day
**33.** How many glasses of water do you drink in a day?	|__| I do not drink water<br>|__| 1 to 2 glasses per day<br>|__| 3 to 4 glasses per day<br>|__| 5 or more glasses per day
**34.** In the last 7 days (1 week), how many days did you eat fish?	|__| I do not eat fish.<br>|__| I did not eat fish in the last 7 days<br>|__| I did eat fish 1 or 2 days in the last week.<br>|__| I did eat fish 3 or 4 days in the last week.<br>|__| I did eat fish 5 or 6 days in the last week.<br>|__| I eat fish every day.<br>|__| I do not remember.
**35.** In the last 7 days (1 week), how many days did you used sweetener or any light/diet product?	|__| I do not use sweetener or any light/diet product<br>|__| I did not used any sweetener or light/diet product in the last 7 days.<br>|__| I did used sweetener or a

	light/diet product 1 or 2 times in the last week.<br>|__| I did used sweetener or a *light/diet* product 3 or 4 times in the last week.<br>|__| I did used sweetener or a *light/diet* product 5 or 6 times in the last week.<br>|__| I did used sweetener or a *light/diet* product every day in the last week.<br>|__| I do not know/ do not remember
40. IN ONE DAY OF a TYPICAL WEEK, how many hours do you use the computer or watch TV or play video game?	|__| I do not do these activities on a typical day week<br>|__| Less than 1 hour a day.<br>|__| About 1 hour a day.<br>|__| About 2 hours a day.<br>|__| About 3 hours a day.<br>|__| About 4 hours a day.<br>|__| About 5 hours a day.<br>|__| About 6 hours a day.<br>|__| About 7 or more hours a day.<br>|__| I do not know/ do not remember

Block 5: Smoking (use of cigarettes or other products that produce smoke)

**41.** Did you ever tried or experimented cigarettes, even one or two puffs?	1. Yes<br>0. No

**42.** How old were you when you tried or experimented cigarettes, even one or two puffs?	0. Never tried<br>1. 9 years old or less<br>2. 10 years old<br>3. 11 years old<br>4. 12 years old<br>5. 13 years old<br>6. 14 years old<br>7. 15 years old<br>8. 16 years old<br>9. 17 years old or more<br>77. I do not know/ do not remember

**43.** Did you ever smoked cigarettes for at least 7 days in a row, in other words, for a full week?	1. I have never smoked<br>2. Yes<br>3. No<br>77. I do not know/ do not remember

**44.** Do you currently smoke?	1. Yes<br>2. No

**45.** IN THE LAST 30 DAYS (one month), how many days did you smoke cigarettes?	0. I have never smoked<br>1. I did not smoke cigarettes in the last month.<br>2. 1 to 2 days.<br>3. 3 to 5 days.<br>4. 6 to 9 days.<br>5. 10 to 19 days.<br>6. 20 to 29 days.<br>7. Every day.<br>77. I do not know/ do not remember

**46.** IN THE LAST 30 DAYS (one month), in the days you smoked,	0. I have never smoked.<br>1. I did not smoke cigarettes in the last 30 days.

how many cigarettes did you smoke on average?	2. Less than 1 cigarette per day.<br>3. 1 cigarette per day.<br>4. 2 to 5 cigarettes per day.<br>5. 6 to 10 cigarettes per day.<br>6. 11 to 20 cigarettes per day.<br>7. 21 to 30 cigarettes per day.<br>8. More than 30 cigarettes per day.<br>77. I do not know/ do not remember

**47.** How old were you when you started smoking daily?	0. I have never smoked<br>1. I have never smoked daily.<br>2. 9 years old or less.<br>3. 10 years old.<br>4. 11 years old.<br>5. 12 years old.<br>6. 13 years old.<br>7. 14 years old.<br>8. 15 years old.<br>9. 16 years old.<br>10. 17 years old or more.<br>77. I do not know/ do not remember

48. Do you smoke flavored cigarettes?

1. Mint, menthol or spearmint?	0. I do not smoke cigarettes.<br>1 Yes.<br>2 No.

2. Clove or Bali?	0. I do not smoke cigarettes.<br>1 Yes.<br>2 No.

3. Vanilla, cream, cherry, strawberry, chocolate or other flavor?	0. I do not smoke cigarettes.<br>1 Yes.<br>2 No.

49. When you started smoking, what kind of cigarettes you smoked more:	0. I have never smoked.<br>1. Mint, menthol or spearmint flavored cigarettes.<br>2 Bali cigarettes, flavored with cloves.<br>3 Vanilla, cream, cherry, strawberry, chocolate flavored cigarettes or other flavor.<br>4 Ordinary cigarettes or

ERICA
ESTUDO DE RISCOS CARDIOVASCULARES EM ADOLESCENTES

	unflavored.

50. Why do you smoke flavored cigarettes? (You can select more than one option).	0 I have never smoked.<br>1 They are tastier.<br>2 They do not irritate the throat.<br>3 They are more charming.<br>4 The packets are more beautiful.<br>5 Other.<br>77. I do not know.

**51.** Do you stay in contact with the smoke of cigarettes (or cigars, pipes, cigarillos) of other people in the house where you live?	1. Yes<br>0. No

**52.** How many days per week do you usually stay in contact with the smoke of cigarettes (or cigars, pipes, cigarillos) of other people in the house where you live?	0. I do not stay exposed to tobacco smoke from other people in the house where I live.<br>1. Less than 1 day.<br>2. 1 to 2 days.<br>3. 3 to 4 days.<br>4. 5 to 6 days.<br>5. Every day.<br>77. I do not know.

53. Besides you, how many people in your family or living with you smoke in the house where you live?	0. No one smokes in the house where I live.<br>1. 1 person.<br>2. 2 to 3 persons.<br>3. 4 or more persons.

54. Do you stay in contact with the smoke of cigarettes (or cigars, pipes, cigarillos) of others outside your home (at school, parties, bars, work or other places) smelling smoke?	1. Yes<br>0. No

55. How old were you when you drink AT LEAST ONE CUP OR A DOSE of alcohol for the first time? Do not consider the times you tasted or drank just a few sips.	0. I have never experimented or drink alcohol.<br>1. I have never experimented or drink alcohol, just a few sips.<br>2. 9 years old or less<br>3. 10 years old<br>4. 11 years old<br>5. 12 years old<br>6. 13 years old<br>7. 14 years old<br>8. 15 years old<br>9. 16 years old<br>10. 17 years old or more<br>77. I do not know/ do not remember

56. In the LAST 30 DAYS (one month), how many days you drank AT LEAST ONE CUP OR A DOSE of alcohol?	0. I have never drink alcohol.<br>1. I did not drink alcohol in the last month<br>2. 1 or 2 days<br>3. 3 or 5 days<br>4. 6 or 9 days<br>5. 10 or 19 days<br>6. 20 or 29 days<br>7. All 30 days<br>77. I do not know/ do not remember

57. In the LAST 30 DAYS (one month), in the days when you drank some alcoholic drink how many cups or doses you took on average?	0. I have never drink alcohol.<br>1. I have not drink alcohol in the last 30 days<br>2. Less than a cup or dose<br>3. 1 cup or 1 dose<br>4. 2 cups or 2 doses<br>5. 3 cups or 3 doses<br>6. 4 cups or 4 doses<br>7. 5 cups or more; 5 doses or more in the last 30 days<br>77. I do not know/ do not remember

58. What kind of alcoholic drink do you consume most often?	1. I do not drink alcohol<br>2. Beer<br>3. Wine<br>4. Ice vodka<br>5. Cachaça or cachaça-based drinks<br>6. Tequila, vodka or rum-based drinks<br>7. Another kind of drink

59. At what age did you have your first period?	0. I did not have my first period yet.<br>1. 9 years old or less.<br>2. 10 years old.<br>3. 11 years old.<br>4. 12 years old.<br>5. 13 years old.<br>6. 14 years old.<br>7. 15 years old.<br>8. 16 years old.<br>9. 17 years old or more.<br>77. I do not know/ do not remember.
60. Do you menstruate every month?	0. I did not have my first period yet.<br>1. Yes.<br>2. No.
61. At what age hair started to appear in your genital area?	0. I do not have pubic hair.<br>1. 9 years old or less.<br>2. 10 years old.<br>3. 11 years old.<br>4. 12 years old.<br>5. 13 years old.<br>6. 14 years old.<br>7. 15 years old.<br>8. 16 years old.<br>9. 17 years old or more.<br>77. I do not know/ do not remember.
62. Have you ever had sexual intercourse?	1. Yes<br>0. No
63. At what age did you have your first sexual intercourse?	0. I never had sexual relations.<br>1. 9 years old or less.<br>2. 10 years old.<br>3. 11 years old.<br>4. 12 years old.<br>5. 13 years old.<br>6. 14 years old.<br>7. 15 years old.<br>8. 16 years old.<br>9. 17 years old or more.<br>77. I do not know/ do not remember.
64. The last time you had sexual relations you or your partner used (you can select more than one option):	0. I never had sexual relations.<br>1. Contraceptive pill.<br>2. Day after pill<br>3. Condom.

ERICA
ESTUDO DE RISCOS CARDIOVASCULARES EM ADOLESCENTES

	4. Other.
65. Do you use contraceptive pill?	1. |__| Yes 0. |__| No
66. Are you pregnant?	1. |__| Yes 0. |__| No

Block 8: Oral health

Do your gums bleed?	1. |__| Yes 0. |__| No
When was the last time you went to the dentist?	0|__| Never<br>1|__| Less than 6 months<br>2|__| 6 months or more<br>77 |__| I do not know/ do not remember
How many times a day, usually, do you brush your teeth?	0|__|I do not brush my teeth.<br>1|__| One time.<br>2|__| Two times.<br>3|__| Three times.<br>4|__| More than three times.
To clean your teeth, do you usually use a toothbrush?	1. |__| Yes<br>0. |__| No
To clean your teeth, do you usually use dental floss?	1. |__| Yes<br>0. |__| No
To clean your teeth, do you usually use toothpaste?	1. |__| Yes<br>0. |__| No

ERICA
ESTUDO DE RISCOS CARDIOVASCULARES EM ADOLESCENTES

67. Have any doctor ever said that you have or had high blood pressure (Hypertension)?	1. |__| Yes 0. |__| No<br>77. |__| I do not know/ do not remember
68. How old were you when a doctor first told you had high blood pressure (Hypertension)?	0.|__| No doctor told me I have or had high blood pressure<br>1.|__| Less than 12 years old.<br>2. |__| 12 years old.<br>3. |__| 13 years old.<br>4. |__| 14 years old.<br>5. |__| 15 years old.<br>6. |__| 16 years old.<br>7. |__| 17 years old or more.<br>77. |__| I do not know/ do not remember
69. Do you take some medication for high blood pressure (Hypertension)?	1. |__| Yes. 0. |__| No.<br>77. |__| I do not know/ do not remember
70. Have any doctor ever said you have high blood sugar (Diabetes)?	1. |__| Yes. 0. |__| No.<br>77. |__| I do not know/ do not remember
71. How old were you when a doctor first told you had high blood sugar (Diabetes)?	0.|__| No doctor told me that I have or had high blood sugar.<br>1.|__| Less than 12 years old.<br>2. |__| 12 years old.<br>3. |__| 13 years old.<br>4. |__| 14 years old.<br>5. |__| 15 years old.<br>6. |__| 16 years old.<br>7. |__| 17 years old or more.<br>77. |__| I do not know/ do not remember
72. Do you take some medication for high blood sugar (Diabetes)?	1. |__| Yes. 0. |__| No.<br>77. |__| I do not know/ do not remember
73. What kind of medicine for high blood sugar (diabetes) do you take?	0.|__| I do not use diabetes medication.<br>1. |__| Pill.<br>2. |__| Insulin.
74. Have any doctor ever said that you have or had high blood lipids (cholesterol or	1. |__| Yes. 0. |__| No.<br>77. |__| I do not know/ do not remember

ERICA
ESTUDO DE RISCOS CARDIOVASCULARES EM ADOLESCENTES

triglycerides)?	
75. How old were you when a doctor first told you had high blood lipids (cholesterol or triglycerides)?	0.|__| No doctor told me that I have or had high blood lipids.<br>1.|__| Less than 12 years old.<br>2. |__| 12 years old.<br>3. |__| 13 years old.<br>4. |__| 14 years old.<br>5. |__| 15 years old.<br>6. |__| 16 years old.<br>7. |__| 17 years old or more.<br>77. |__| I do not know/ do remember
76. In the LAST 12 MONTHS (one year), how many wheezing crisis have you had?	0. |__| I have never had wheezing crisis.<br>1. |__| I have not had wheezing crisis in the last 12 months.<br>2. |__| 1 to 3 crisis<br>3. |__| 4 to 12 crisis<br>4. |__| More than 12 crisis<br>77. |__| I do not know/ do remember
77. Have any doctor ever said you have asthma?	1. |__| Yes. 0. |__| No.<br>77. |__| I do not know/ do not remember
78. Are you satisfied with your body weight?	1. |__| Yes 0. |__| No
79. In your opinion what is your current body weight:	1. |__| Below the ideal.<br>2. |__| Ideal.<br>3. |__| Above the ideal.<br>4. |__| High above the ideal.
80. How would you wish your body weight to be?	1. |__| I am satisfied with my body weight.<br>2. |__| Lower than it is.<br>3. |__| Much Lower than it is.<br>4. |__| Above than it is.<br>5. |__| Much above than it is.

87. In ONE DAY OF TYPICAL WEEK, at what time do you usually sleep?

6:00 P.M.	6:00 A.M.
7:00 P.M.	7:00 A.M.
8:00 P.M.	8:00 A.M.
9:00 P.M.	9:00 A.M.
10:00 P.M.	10:00 A.M.
11:00 P.M.	11:00 A.M.
Midnight	noon
1:00 A.M.	1:00 P.M.
2:00 A.M.	2:00 P.M.
3:00 A.M.	3:00 P.M.
4:00 A.M.	4:00 P.M.
5:00 A.M.	5:00 P.M.

88. In ONE DAY OF TYPICAL WEEK, at what time do you usually wake up?

4:00 A.M.	4:00 P.M.
5:00 A.M.	5:00 P.M.
6:00 A.M.	6:00 P.M.
7:00 A.M.	7:00 P.M.
8:00 A.M.	8:00 P.M.
9:00 A.M.	9:00 P.M.
10:00 A.M.	10:00 P.M.
11:00 A.M.	11:00 P.M.
noon	Midnight
1:00 P.M.	1:00 A.M.
2:00 P.M.	2:00 A.M.
3:00 P.M.	3:00 A.M.

89. In weekends, at what time do you usually sleep?

6:00 P.M.	6:00 A.M.
7:00 P.M.	7:00 A.M.
8:00 P.M.	8:00 A.M.
9:00 P.M.	9:00 A.M.
10:00 P.M.	10:00 A.M.
11:00 P.M.	11:00 A.M.
Midnight	noon
1:00 A.M.	1:00 P.M.
2:00 A.M.	2:00 P.M.
3:00 A.M.	3:00 P.M.
4:00 A.M.	4:00 P.M.
5:00 A.M.	5:00 P.M.

90. In weekends, at what time do you usually wake up?

4:00 A.M.	4:00 P.M.
5:00 A.M.	5:00 P.M.
6:00 A.M.	6:00 P.M.
7:00 A.M.	7:00 P.M.
8:00 A.M.	8:00 P.M.
9:00 A.M.	9:00 P.M.
10:00 A.M.	10:00 P.M.
11:00 A.M.	11:00 P.M.
noon	Midnight
1:00 P.M.	1:00 A.M.
2:00 P.M.	2:00 A.M.
3:00 P.M.	3:00 A.M.

101. What did you find about this questionnaire?	1|__| Very easy to answer<br>2 |__| Easy to answer<br>3 |__| Neither easy nor difficult to answer<br>4 |__| Difficult to answer<br>5 |__| Very difficult to answer
